# Cross-Cultural Validity and Reliability of the Questionnaire on Back-Health-Related Postural Habits During Daily Activities in the Polish Young Adolescent Population

**DOI:** 10.3390/jcm14217793

**Published:** 2025-11-03

**Authors:** Manuel Monfort-Pañego, Marta Kinga Labecka, Vicente Miñana-Signes, Agnieszka Jankowicz-Szymańska

**Affiliations:** 1 Teacher Education in Physical Education Department, Teacher Education Faculty, University of Valencia, 46022 València, Spain; manuel.monfort@uv.es; 2 Faculty of Rehabilitation, Jozef Pilsudski University of Physical Education, 00-968 Warsaw, Poland; 3 IES Ferrer i Guàrdia, Conselleria d’Educació, 46015 València, Spain; 4 Department of Physiotherapy, Faculty of Medicine and Health Sciences, University of Applied Sciences in Tarnow, 33-100 Tarnow, Poland

**Keywords:** aged, habits, posture, reproducibility of results, validation study

## Abstract

**Background/Objectives:** This study aimed to conduct the cross-cultural adaptation and assess the reliability of a validated measurement instrument, the Back-Health Related Postural Habits in Daily Activities (BEHALVES). **Methods:** Following a standardized methodological framework for the cross-cultural adaptation of health-related instruments, the BEHALVES questionnaire was translated and tested in 104 primary education students (mean age 13.8 ± 0.74 years; 49.1% girls) at two different time points with a 1-week interval between each test. The analysis was expressed as test–retest reliability according to the differences observed between the readings (T1–T2, α = Cronbach’s alpha), the standard deviation of the differences (SD), the intraclass correlation coefficient (ICC), 95% confidence intervals for the intraclass correlation coefficient, standard error of measurement (SEM), coefficient of repeatability (CR), and minimal detectable change (MDC). **Results:** Internal consistency results for the joint analysis of items grouped into categories were good (α = 0.72). Lying down was the worst-performing category in this study. The ICC was excellent for the total score (0.96) and all categories (0.90–0.99). Regression analysis between the mean of the two measures and their difference indicated that there was a significant difference (F1,103 = 0.19, *p* < 0.001; beta coefficient = −0.15, *p* < 0.001; R2 = 0.16). **Conclusions:** The BEHALVES questionnaire is valid and reliable for the Polish youth population, providing similar results to the original Spanish version. The continued development of reliable measurement instruments will enhance research in education, public health, and clinical practice, ultimately leading to improved strategies for preventing postural issues in young populations.

## 1. Introduction

Correct posture is widely regarded as an indicator of overall health. For several decades, scientific literature has provided evidence supporting this claim [1,2,3]. However, defining correct posture is challenging due to variations in body shape influenced by biological sex and ethnicity [4]. Some researchers define correct posture as the alignment of body segments in a standing position that optimizes balance and minimizes muscular effort [5]. Others emphasize its relationship with physiological, occupational, and biomechanical factors [6]. Despite these differing definitions, scholars universally acknowledge the correlation between poor posture and a diminished quality of daily functioning, which may result from an increased risk of chronic pain and degenerative changes [7], as well as impairments in cardiorespiratory, digestive, concentration, and cognitive functions [8].

During adolescence, the body undergoes rapid growth and musculoskeletal adaptations that influence balance, coordination, and spinal alignment [5,6]. Maintaining correct posture at this stage is essential to prevent functional asymmetries, spinal deformities, and musculoskeletal pain later in life [7,9]. Numerous studies report a high prevalence of postural deviations among young populations worldwide, ranging from 50% to 80%, particularly among adolescents with obesity [10,11,12,13,14]. Contributing factors include prolonged sitting, excessive use of digital devices, carrying heavy school bags, insufficient physical activity, and poor ergonomic conditions in schools and homes [5,12,13,14,15].

Physical activity (PA) plays a crucial role in maintaining spinal health. Although some studies have not found a direct association between PA levels and postural dysfunctions [16], engaging in regular, moderate activity enhances the recruitment of postural muscles—such as the erector spinae, multifidus, and transversus abdominis—which contribute to spinal stability and proper alignment [17,18,19,20,21]. Consequently, sufficient PA may help prevent or correct postural abnormalities and protect against conditions such as scoliosis or low back pain [22]. Nevertheless, according to a WHO report, nearly 80% of adolescents fail to meet the recommended levels of physical activity [23].

Although young adults (18–21 years old) generally possess good knowledge of posture—demonstrating an ability to define correct posture and recognize its importance for health and fitness—fewer than 30% express willingness to participate in an exercise program aimed at improving postural habits [7]. Similar findings were reported by Montuori et al. [9], who analyzed the knowledge, attitudes, and behaviors related to correct body posture in a sample of 1177 adults. Despite their awareness and positive attitudes toward posture, only 2.8% of participants had consulted a specialist for postural evaluation and improvement. Both studies highlight that individuals with greater knowledge tend to exhibit better posture-related behaviors, with age and education also playing significant roles.

Childhood is the optimal period for establishing healthy lifestyle habits [24]. Therefore, there is a clear need to implement enhanced educational and training programs to promote correct posture as an essential component of well-being. However, the development of such programs requires a preliminary analysis of the strengths and weaknesses in children’s and adolescents’ knowledge, attitudes, and behaviors related to posture.

Several instruments have been developed to assess posture-related knowledge and behaviors in young populations [25,26], such as the Back Health Knowledge Questionnaire (BHKQ) [27], the Back Pain and Body Posture Evaluation Instrument (BackPEI) [28], and the adult version of the Aristegui questionnaire [29]. However, these instruments mainly focus on posture-related knowledge and ergonomic awareness rather than habitual postural behaviors in daily life. The Back-Health-Related Postural Habits in Daily Activities Questionnaire (BEHALVES) [30] was specifically designed to assess postural behaviors during daily activities and has demonstrated good internal consistency and test–retest reliability in its original Spanish validation. Currently, no tools with proven reliability and validity exist for this purpose in Poland. Accordingly, this study addressed the following research question: Is the Polish version of the BEHALVES questionnaire a reliable and culturally valid tool for assessing postural habits among young adolescents?

We hypothesized that the adapted version would demonstrate good internal consistency and excellent test–retest reliability, comparable to those reported for the original Spanish instrument [30].

## 2. Materials and Methods

### 2.1. Study Design

The study followed the cross-cultural adaptation framework proposed by Sousa and Rojjanasrirat [31], which involves forward and backward translation, expert review, pilot testing, and psychometric evaluation. This systematic process ensured conceptual and cultural equivalence between the original and Polish versions of the BEHALVES questionnaire.

### 2.2. Ethical Statement

The study was approved by the Bioethics Committee of the University of Applied Sciences in Tarnow (no. 21/2024; date: 10 July 2024) and conducted in accordance with the Declaration of Helsinki. Written informed consent was obtained from students, parents, and the headmaster.

### 2.3. Instrument

The Back-Health-Related Postural Habits in Daily Activities (BEHALVES) tool was cross-culturally adapted and reliability tested [30]. BEHALVES was developed to study daily body postural habits in adolescents. The items were divided into categories: standing posture (items 1–4), sitting posture (items 5–13), using backpacks (items 14–20), lifting weights (items 21–26), and lying posture (items 27–31). The items in the questionnaire were scored with a 4-point Likert scale: 1 = Never, 2 = Hardly ever, 3 = Almost always, and 4 = Always [30]. The authors obtained permission from the copyright holders to use this tool [30].

### 2.4. Translation and Cross-Cultural Adaptation

Three translators (native speakers), four researchers (authors of this study), and physiotherapists organized and facilitated the translation process. The BEHALVES questionnaire was translated and cross-culturally adapted from the original Spanish version into Polish in accordance with the established guidelines [31], consisting of several steps.

Step one: A bilingual Spanish-speaking translator performed the translation (from Spanish into Polish). The translator aimed to retain the concepts of the original while using culturally fitting expressions. The translator was given a clear explanation of the concepts to capture the conceptual meaning of the items while keeping the language colloquial.

Step two: A professional, bilingual, native-speaking Spanish translator carried out a back-translation (from Polish to Spanish) without knowing the original version of the questionnaire. This stage aimed to detect discrepancies between the source document and the translation.

Step three: The person fluent in Polish living in Valencia compared the target questionnaire with the original version (from Spanish to Polish). This stage aimed to detect possible differences between the backward and the original versions and improve these through consensus among the translators. Additionally, the principal researcher reviewed these translations and, with the help of the translators, made sure the Polish version reflected the same item content as the original version and was conceptually equivalent.

Step four: To harmonize the questionnaire adaptation process, an expert committee participated in the revisions of the questionnaire. The committee consisted of four experts: two physiotherapists and two physical educators (doctors, professors, physiotherapists, PE teachers, and instructors of corrective gymnastics/exercises; Polish and Spanish). To identify any discrepancies or errors, the committee examined the semantic (i.e., equivalence in the meaning of words), idiomatic (i.e., equivalence in idioms and colloquialisms), experiential (i.e., equivalence in the target cultural context), and conceptual (i.e., equivalence of the concept and the experiences of the target culture) of the items and response options. This phase ended after agreement on a pre-final version.

Step five: The expert committee performed a test of the pre-final version of the questionnaire aimed to identify the level of acceptability, interpretation, comprehensibility, relevance of the adaptation, and any other issues that cause uncertainty, such as ambiguous wording, overlapping or redundant items, or difficulties in understanding or choosing among response options. Physiotherapy students answered the following questions: (1) Was the questionnaire easy to complete? (2) Were the instructions clear to you? (3) Were the answer options clear to you? (4) Were there any questions that you found difficult? (5) Were there any irrelevant questions? The expert committee reviewed the answers to identify any important changes for improving the Polish pre-final questionnaire version.

Step six: All proofreading and typographical errors were corrected before producing the final Polish version (Supplementary Materials Files S1 and S2).

### 2.5. Participants

The questionnaire was conducted on a sample of the target population to analyze the reliability of the translated instrument. The sample consisted of 116 students of upper primary education by convenience sampling from a public school in the east of Poland. Twelve participants were excluded because (1) they did not complete the second questionnaire, (2) they did not consent to participate in the study, or (3) they were not present at school. The final sample was 104 students (89.7% recruited; 13.8 ± 0.74 years old; 49.1% girls, *n* = 51) who participated in the study. The sample size was determined in accordance with the methodological recommendations for instrument validation studies, which propose a minimum of 100 participants or at least five to ten respondents per item to ensure a reliable estimate of the psychometric properties [32]. The age range (12–15 years) corresponds to the available primary school population in grades 6–8 of Polish primary education. Older adolescents (16–18 years) were not included due to access limitations within secondary education. The distribution of the study sample and their scores are presented in Table 1.

### 2.6. Procedure

This study was carried out between September 2024 and November 2024. The questionnaire was undertaken under the supervision of one member of our research group during class time with a teacher. The questionnaire was prepared in paper form. The aim of the study was presented to the students, then the questions were read out one by one, any questions from the participants were answered, and the procedure for filling out the form was explained (T1). The questionnaires were administered for the second time (T2), with a 1-week interval between each test [33] to evaluate the test–retest reliability. The authors chose this period based on the concept that the time should be long enough for respondents not to remember previously selected answers but short enough that they are unlikely to assimilate new knowledge [33]. At the second time (T2), the same questionnaire was administered. The students took 10–20 min to complete the questionnaire according to their age.

At the beginning of the questionnaire, students had to indicate that they voluntarily and knowingly consented to participate in the study and to use the research results in scientific publications and conferences. Each student received an ID to maintain the anonymity of the data. To guarantee the precision and validity of the data transcription to Excel, an independent researcher applied a double entry of the data [34]. Since the risk in this data type is low, it was applied to a sample of 20% of the test data from the randomly chosen participants. After collecting the data, one research group member entered it into Microsoft Excel.

### 2.7. Data Analysis

The study analyzed item scores, mean scores for each category, and the overall mean score, measured at two different times. SPSS version 28 (IBM Corp., Armonk, NY, EE. UU.). and Microsoft Excel version 16.89.1 for Mac (Microsoft Corporation, Redmond, Washington, EE.UU.) were used for data analysis. The items were transformed and grouped into categories of standing, sitting, carrying, heavy lifting, and lying habits. The final data analysis setup used in previous studies was applied [30].

To determine the inter-rater reliability between the measures at T1 and T2, the differences between the mean scores and the mean of the two BEHALVES questionnaire scores were calculated. They were plotted using the Bland–Altman plot (95% agreement limit) [7].

The psychometric analysis was expressed as test–retest reliability according to the differences observed between the readings (T1–T2), the standard deviation of the differences, and the intraclass correlation coefficient (ICC) [35], 95% confidence intervals for the ICC [36], standard error of measurement (SEM), coefficient of repeatability (CR), and minimal detectable change (MDC) [37].

A regression analysis was applied to study the interaction between measurement error and the total score of the BEHALVES questionnaire [7].

Repeated measures analysis of variance was used to assess the effect of time, gender, and age between and within subjects (T1, T2) on total scores.

A one-factor analysis of variance between the scores of the different quartiles was used to study the discriminatory power of the test. Floor/ceiling effects were analyzed by calculating the percentage of adolescents scoring in the highest and lowest quartiles of the total score of Test 1. If >15% of participants scored in the lowest/highest quartile, a floor/ceiling effect was considered to exist [35].

## 3. Results

### 3.1. Translation and Cross-Cultural Adaptation

The translation procedure took approximately one month to achieve a culturally appropriate version, and all the items were easily translated forward and backward. No difficulties were evidenced during the forward and backward translations of the questionnaire. Some concern was raised about the difficulty in discriminating between the terms “turn” versus “rotate torso” and “folder” versus “briefcase/briefcase without a handle”.

The expert committee revised the questionnaire in three rounds to finalize the definitive version. In the question about the back position during household chores, the Spanish version included “ironing”. In contrast, in the Polish version, it was removed because a primary school child is unlikely to perform this type of activity at home. Then, the answers to the question about the type of bag usually used to carry books and school supplies were modified. The answer “folder” was changed to “briefcase/briefcase without handles”, and the answer “backpack in the form of a suitcase” was removed because it previously had the same meaning as “backpack on wheels”.

The question about wearing high-heeled shoes while standing was incomprehensible. The question was modified to “I experience back pain when I walk for a long time in high-heeled shoes. This question was only for people who regularly wear high-heeled shoes. If this question does not apply to you, go to the next point”. However, the meaning of the question thus changed: children were not asked about wearing habits of high heels, but whether they experienced pain when wearing them. Thus, the researcher who calculated the results gave the maximum value to those who had not answered because they did not wear high heels and coded the other results according to whether the pain was less or more severe.

Finally, the expert panel added the last section of the questionnaire, the “opinion on the questionnaire”. These were three questions regarding the evaluation of the graphics used in the questionnaire, the comfort of completing the questionnaire, and the incomprehensibility of any of the questions. These questions were not analyzed for reliability. As no other significant problems or grammatical or spelling errors were identified, the expert committee confirmed the work performed and finalized the last version of the questionnaire.

### 3.2. Reliability of the Questionnaire

Internal consistency results for the joint analysis of items grouped into categories were good (Cronbach’s α = 0.72). Lying down was the worst-performing category in this study.

The results of the psychometric study with the test–retest values are shown in Table 2. The mean differences between test and retest were not different from zero for the mean total scores. Only the mean difference for the mobilizing heavy weights category differed from zero (*p* = 0.001). The intraclass correlation coefficient (ICC) was excellent for the total score (0.96) and all categories (0.90–0.99). The low values of SEM (0.02) and MDC (0.05) were good for the total score and for each category with an adjusted CR value (Table 2).

**Figure 1:**
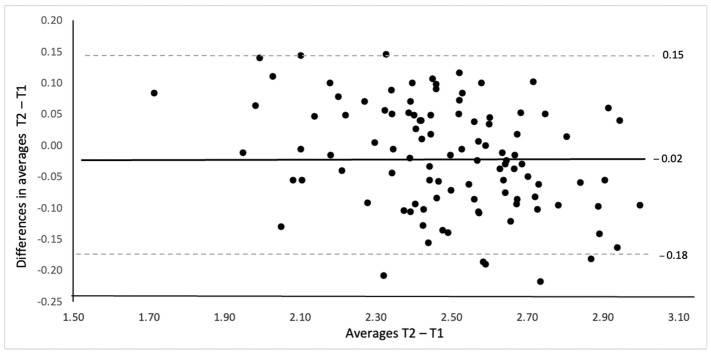
Bland–Altman plot for absolute mean scores of BEHALVES. The solid line represents the mean difference (−0.02 points), and dashed lines represent the 95% limits of agreement (−0.18 to 0.15 points). T1 = time 1, T2 = time 2.

**Figure 2:**
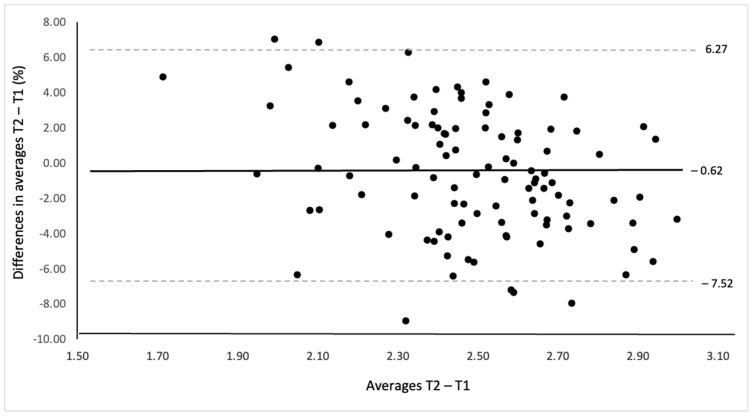
Bland–Altman plot for relative mean scores of BEHALVES. The solid line represents the mean difference (−0.62%), and dashed lines represent the 95% limits of agreement (−7.25% to 6.27%). T1 = time 1, T2 = time 2.

Figure 1 and Figure 2 show the results of the Bland–Altman plot for the mean total score. The absolute and relative values of mean differences (−0.02, −0.62%) and the limits of agreement (−0.18 and 0.15 points) indicated a measurement error of less than 1%.

Regression analysis between the mean of the two measures and their difference indicated that there was a significant difference (F1,103 = 0.19, *p* < 0.001; beta coefficient = −0.15, *p* < 0.001; R^2^ = 0.16).

Repeated measures ANOVA indicated that there were no significant differences within total means of time 1 and 2 (F1,100 = 3.002, *p* = 0.086), time × sex (F1,100 = 0.91, *p* = 0.34), time × age (F1,100 = 0.30, *p* = 0.86), or time × sex × age (F1,100 = 1.39, *p* = 0.24), and there were no significant between-subject contrasts.

The study on the discriminative ability of the test indicated that the scores between all quartiles of the sample were significantly different (F3,100 = 183.34; *p* < 0.001). The quartile-to-quartile comparison showed significant differences between all quartiles, indicating a high degree of sensitivity of the test (*p* < 0.001). Only one participant scored in the first quartile of the score range (1.71 points) and none in the last quartile, confirming that the floor/ceiling effect did not occur.

The average time each participant took to complete the questionnaire was 16.02 min (SD ± 4.32) for T1 and 12.8 min (SD ± 3.27) for T2. No floor/ceiling effects were observed in the total score of T1 (0% floor/ceiling). On a scale from 1 to 3, 8% of participants were in the 1–2 score range, and 4.2% of participants were in the 3–4 score range.

The solid line marks the difference to the overall average in relative value (−0.62%). Dashed lines mark limits of agreement within ±1.96 standard deviations (6.27%, −7.25%).

### 3.3. Opinion of the Questionnaire

Respondents were invited to give their opinions on the questionnaire and their difficulties in understanding the questions and answers, as well as to comment on other aspects.

The average rating of the graphics used in the questionnaire was 6.8 in the first round of testing and 8.0 in the retest. Children were asked to mark one value on a linear scale from 1 = Illegible, not very helpful to 10 = Legible, very helpful.

On the other hand, the average rating of the comfort of completing the questionnaire improved from 5.5 for the first time to 3.3 for the second time. Children were asked to mark one value on a linear scale from 1 = The questionnaire does not take much time and filling it out is interesting to 10 = Filling out the questionnaire takes a lot of time and is tiring.

Only when the children were completing the questionnaire for the first time did they report that questions 2.5 and 4.2 and the word “fetal position” were incomprehensible. These questions were not analyzed for reliability, but they helped validate the questionnaire.

This section provides a concise description of the experimental results, their interpretation, and the conclusions that can be drawn from them.

## 4. Discussion

This study aimed to perform the cross-cultural adaptation and assess the reliability of a validated measurement instrument (BEHALVES). The Polish version of the BEHALVES questionnaire proved to be reliable and yielded results consistent with the original Spanish version, making it relevant for epidemiological research in the studied population.

### 4.1. Cross-Cultural Validation

Cross-cultural adaptation of measurement instruments is essential to ensure that a tool maintains its psychometric properties when applied in different linguistic and cultural contexts [38]. This process involves not only translation but also cultural adaptation to preserve the conceptual meaning of items. In this study, the translation process was rigorous, with forward and backward translations followed by expert committee review. Adjustments, such as modifying items related to household chores and school supplies, ensured the questionnaire remained culturally relevant for Polish youth while maintaining conceptual equivalence.

The importance of cross-cultural validation is particularly relevant in health-related research [39], where differences in language, education systems, and lifestyle factors can influence participants’ understanding and responses. The inclusion of an opinion section in this study provided direct feedback from respondents, helping to refine the instrument and confirm its appropriateness for the target population. Given the scarcity of validated instruments assessing postural habits in daily life, the successful adaptation of BEHALVES represents a significant contribution to the field by providing a reliable and culturally sensitive tool.

### 4.2. Reliability and Psychometric Properties

The results of the study confirm that this questionnaire is valid and reliable for use in the Polish youth population. The internal consistency study indicated good reliability (α = 0.72), although its index was slightly less than 0.1 points lower than the alpha of the original Spanish questionnaire [30]. The difference in alpha concerning the original study was minimal; this may be because the study population was slightly smaller (64 fewer participants), and the 16–18 age group was not included in this study.

As in the original study [30], the pattern of the questionnaire scores was unidimensional, as a principal components analysis did not provide better information on the variables analyzed. In this study, we provide item data averaged by conceptual category (Table 2) to show the different scores in each of them, but the key statistical analysis is the total average.

The psychometric study showed a stable pattern of data over time, both for the total mean and for the different categories. These results were even better than those obtained in the original validation study [30]. The first- and second-time values were not significantly different, except for the weightlifting category. This result was the same in the study that validated this questionnaire. The possible reason could be a set of items that assessed unusual movements at these ages, such as lifting heavy weights; however, this is an action to be considered due to the existing evidence of its dangerousness [25,35].

The measurement error data, limits of agreement (Figure 1 and Figure 2), and reliability values (Table 2) indicated an excellent functioning of the questionnaire. The high intraclass correlation coefficients and low standard error of measurement rates reinforced this stability and agreement of the measures. These are the quantitative reasons why this is currently the most solid questionnaire for evaluating postural habits for back health in daily life.

The demands placed on the questionnaire demonstrated its effectiveness in this age group [30]. Although the review study by Ramos et al. [40] noted the existence of other validated instruments, none of them assessed postural habits in daily life. Therefore, a convergent validity analysis was not possible.

While the results indicate that the measures are stable over time, the variation in error based on the mean category scores is an indicator of the study sample’s behavior across different types of tasks.

The regression study showed that as participants’ mean scores increased, their measurement error decreased significantly, reaching zero at the midpoint of the scale (2.5 points) and negative values at high mean scores. This significant relationship has also been described in previous studies [30]. Although their significance was ruled out because of their small effect size (in this case, only 16% of the variance could be due to the relationship between these variables), it is considered interesting to analyze these results.

The negative relationship indicates that at the extreme mean scores, the differences between the measures were larger and inverse. Participants with worse habit scores the first time managed to improve on the second pass, while participants with a better average habit score the first time got worse the second time. The negative and significant trend could indicate that the sample has learned during the period between the first and second pass. However, by crossing the trend line of the scores on the ordinate axis and changing sign, we can say that this has not occurred in this way.

Although it would be ideal to obtain a single value for the differences from the means, the results confirm that those with worse scores have improved and those with better scores have worsened over time. The cause of this result is probably intrinsic to this type of instrument, in which participants’ perceptions vary slightly over time without any action being taken on the issue. Despite this pattern of performance, most of the study sample was found at the borderline of score agreement, where differences have not changed significantly.

The results of this study not only point to its validity and reliability over time but also confirm that the instrument is reliable regardless of the age and sex of the participants. While the Spanish version of this instrument confirmed its reliability between different sexes, in this study, the analysis was extended to the age factor, and its reliability has also been demonstrated. In this regard, it should be noted that the analysis was carried out by grouping the participants into two age groups (12–13 and 14–15), so it would be desirable in future studies to include the ages 16–18 years and, if possible, to achieve sufficient representation to analyze according to the different ages, as it remains to be determined whether the understanding and good performance are masked by the mixture of different ages.

Values related to the minimum detectable change indicated a high sensitivity of the instrument to changes in scores. In this version of BEHALVES, the sensitivity of the instrument was higher than that shown in previous studies [30]. Thus, while a change of 0.37 points can be considered a real change in the Spanish version, in this version, a change of 0.05 points in the overall score can be considered a real change in the study population. This measure is very useful in practical daily use, as patients or students analyzed longitudinally will be able to obtain baseline information on their overall development.

### 4.3. Potential Uses in Different Fields

The successful validation of BEHALVES in Poland demonstrates its potential for application in various fields:

Educational Settings: The instrument can be integrated into school health programs to assess students’ postural habits and inform targeted interventions to promote healthy back practices. Teachers and school nurses can use the data to design preventive measures and ergonomic adjustments in classrooms.

Public Health and Epidemiological Research: The questionnaire provides a standardized method for studying postural health in adolescents across different populations, facilitating international comparisons and cross-cultural studies on back health.

Clinical and Rehabilitation Contexts: Healthcare professionals can use the instrument to monitor postural habits in young patients and develop personalized recommendations for posture correction and injury prevention.

Occupational Health: While primarily designed for students, the methodology behind BEHALVES could be adapted for assessing postural habits in different professional environments, particularly those involving prolonged sitting or heavy lifting.

### 4.4. Limitations and Future Lines

This study presents several limitations that should be acknowledged. First, the sample included only early and middle adolescents (12–15 years), which restricts the generalizability of the findings to older age groups. Second, the study did not include a preregistered protocol. Although preregistration is not mandatory for validation studies, it enhances methodological transparency and limits potential analytical flexibility. Third, the present study did not include a new construct validity analysis, as the factorial structure of the BEHALVES questionnaire was previously examined in the original Spanish validation but could not be confirmed, leading to its interpretation as a unifactorial instrument. Additionally, no other validated instruments currently exist to assess postural habits in daily activities, which made a convergent validity analysis unfeasible. Future research should therefore aim to contrast BEHALVES results with objective indicators of posture and physical activity to expand its psychometric evidence. Fourth, the administration of the questionnaire under researcher supervision in a classroom environment may have introduced a social desirability bias, as participants might have felt inclined to provide responses perceived as correct or desirable. Although the anonymity of responses was emphasized, this potential influence should be considered when interpreting the results.

Finally, some participants initially reported minor difficulties in understanding specific terms (e.g., “fetal position” or “high-heeled shoes”). These issues were resolved through expert review and linguistic clarification during the adaptation process, ensuring that the final Polish version was fully comprehensible for the target age group. Future adaptations might benefit from further cognitive interviews with participants to confirm full semantic equivalence and comprehension across items.

Future research should work on the development of instruments that involve the construction of questions that provide participants with a clearer and more stable understanding of their experiences. To make further progress in research on back health education in the social and educational context, it is necessary to increase the number of studies that develop and validate instruments that are different from the existing ones and that address the specificities in these fields. Research into the diversity and improvement of measuring instruments will make it possible to address methodologically essential problems, such as those of convergence validity. This is a route that must be encouraged for the strengthening of evidence-based research [41] and to make progress in a field that has yet to be explored.

## 5. Conclusions

This study confirms the cross-cultural validity and reliability of the BEHALVES questionnaire for assessing postural habits in Polish youth. While internal consistency and test–retest reliability were confirmed, construct validity (including dimensionality) was not examined here and should be established in future research. The rigorous adaptation process ensured conceptual equivalence with the original Spanish version while maintaining its psychometric robustness.

The findings underscore the need for continued research into culturally adapted measurement tools for back health assessment.

By establishing a validated tool for evaluating postural habits, this study contributes to a growing body of evidence supporting the promotion of back health among adolescents. The continued development of reliable measurement instruments will enhance research in education, public health, and clinical practice, ultimately leading to improved strategies for preventing postural issues in young populations.

## Figures and Tables

**Table 1:** Mean scores of the sample by time, sex, and age.

Time	Sex	Age/Years	Mean Score ± SD	*n*
T1	M	12–13	2.50 ± 0.26	20
		14–15	2.51 ± 0.21	33
		Total	2.50 ± 0.22	53
	F	12–13	2.47 ± 0.29	15
		14–15	2.40 ± 0.31	36
		Total	2.42 ± 0.30	51
	Total T1	12–13	2.48 ± 0.27	35
		14–15	2.44 ± 0.27	69
		Total	2.46 ± 0.27	104
T2	M	12–13	2.46 ± 0.21	20
		14–15	2.49 ± 0.18	33
		Total	2.48 ± 0.19	53
	F	12–13	2.47 ± 0.30	15
		14–15	2.38 ± 0.25	36
		Total	2.41 ± 0.26	51
	Total T2	12–13	2.46 ± 0.25	104
		14–15	2.43 ± 0.22	69
		Total	2.44 ± 0.23	104

Notes: T1 = time 1; T2 = time 2; M = men; F = female; SD = standard deviation.

**Table 2:** Test–retest reliability of the back health questionnaire for category postural habits in daily activities of young students (*n* = 104).

Categories	M ± SD T1	M ± SD T2	M ± SD T1–T2	M ± SD Dif T2–T1	CC	ICC (95%IC)	CR	SEM	MDC
Total	2.50 ± 0.26	2.48 ± 0.23	2.49 ± 0.24	−0.02 ± 0.09	0.94	0.96 (0.95–0.98)	0.17	0.02	0.05
Standing	2.89 ± 0.43	2.86 ± 0.42	2.88 ± 0.41	−0.03 ± 0.21	0.88	0.93 (0.90–0.96)	0.42	0.06	0.16
Sitting	2.41 ± 0.43	2.45 ± 0.36	2.43 ± 0.38	0.04 ± 0.19	0.90	0.94 (0.91–0.96)	0.38	0.05	0.13
Transporting	2.67 ± 0.38	2.69 ± 0.31	2.68 ± 0.34	0.02 ± 0.15	0.93	0.95 (0.93–0.97)	0.29	0.03	0.09
Moving weight	2.22 ± 0.50	2.08 ± 0.47	2.15 ± 0.48	−0.14 * ± 0.16	0.95	0.97 (0.96–0.98)	0.31	0.03	0.08
Lying	2.29 ± 0.57	2.31 ±0.55	2.30 ± 0.56	0.03 ± 0.12	0.98	0.99 (0.98–0.99)	0.24	0.01	0.03

M = mean, T1 = time 1, T2 = time 2, SD = standard deviation, Dif = difference, CC = correlation coefficient, ICC = intraclass correlation coefficient, CR = coefficient of repeatability, SEM = standard error of measure, MDC = minimum detectable change. * Significative differences *p* < 0.01.

## Data Availability

The original contributions presented in this study are included in the article/supplementary material. Further inquiries can be directed to the corresponding author(s).

## Supplementary Materials

The following supporting information can be downloaded at https://www.mdpi.com/article/10.3390/jcm14217793/s1, File S1: English-translated version of the BEHALVES questionnaire. File S2: Polish validated version of the BEHALVES questionnaire.
