# Supplementary material for: Cross-Cultural Validity and Reliability of the Questionnaire on Back-Health-Related Postural Habits During Daily Activities in the Polish Young Adolescent Population
Source: J Clin Med. 2025 Nov 3;14(21):7793. doi: 10.3390/jcm14217793 (PMC12608106; doi:10.3390/jcm14217793)
Supplement: Supplementary file 1 [file jcm-14-07793-s001.zip › File S1.pdf]

AGE ……………………..

I voluntarily give my informed consent to participate in the survey and for the results of the study to be presented at conferences and in scientific publications

O I AGREE **O I DISAGREE**

BACK CARE AND POSTURAL HABITS QUESTIONNAIRE

MARK WITH THE X THE ANSWER YOU THINK IS MOST APPROPRIATE

1. When I'm standing:

		Never	Almost never	Almost always	Always
1.1.	When standing and brushing my teeth, I support myself with my free hand to relieve the strain on my bent spine. 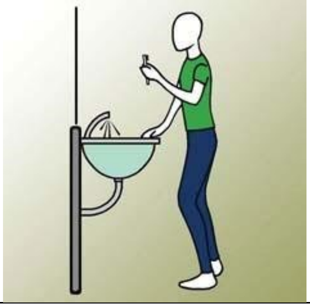				
1.2.	When standing for a long time, I constantly change position, shifting my body weight from leg to leg.				
1.3.	When standing and doing household chores (sweeping, making the bed) I try to keep my back straight. 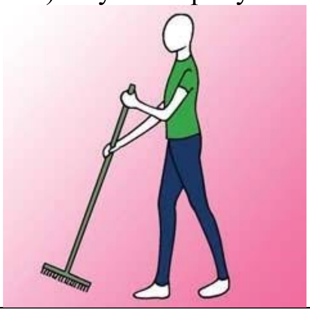				
1.4.	*This question is addressed only to people who regularly walk in high-heeled shoes. If this question does not apply to you, move on to the next section.* I have back pain when walking for a long time in high-heeled shoes.				

2. When I'm sitting:

		Never	Almost never	Almost always	Always
2.1.	When sitting, I lean my whole back against the back of the chair. 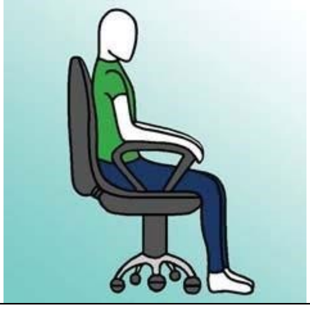				
2.2.	When sitting, I do not rest both feet on the ground. 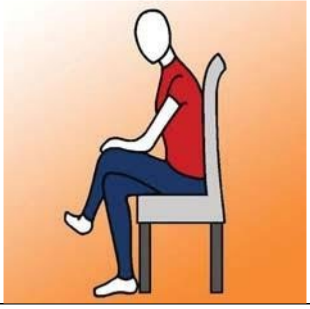				
2.3.	When sitting, I rest my buttocks on the edge of the seat. 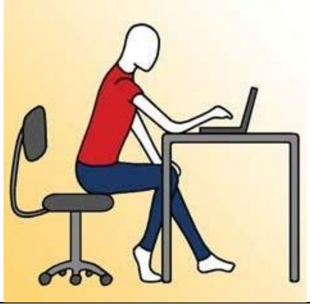				
2.4.	When sitting, I lean forward and arch my back. 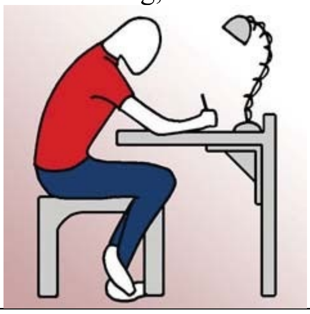				
2.5.	When sitting, I turn my torso to pick up an object or talk to my classmates.				

	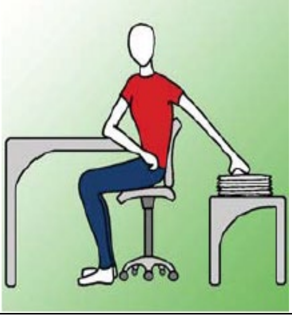				
2.6.	When sitting, I rest my forearms on the table top. 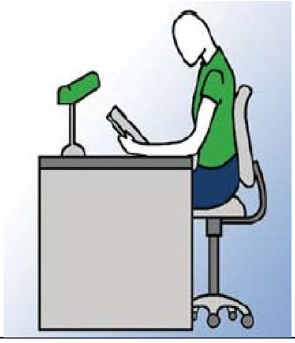				
2.7.	When sitting, I prop up a book or tablet on a stand. 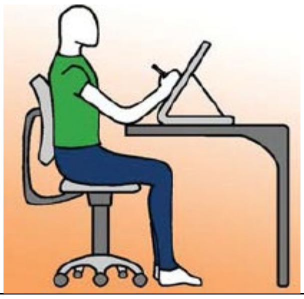				
2.8.	When working on the computer, I have the screen at eye level. 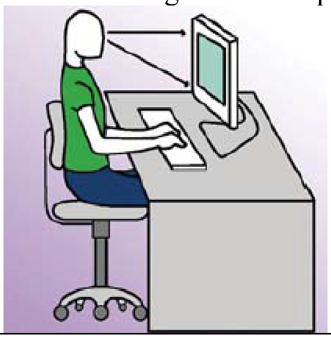				
2.9.	When working on the computer, I put the keyboard and the mouse at elbow height. 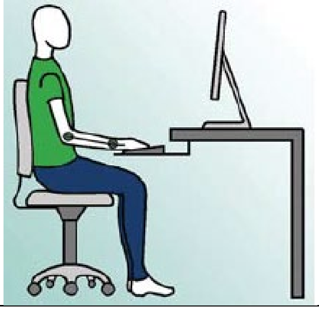				

3. WHEN I'M CARRYING HEAVY OBJECTS:

3.1. What kind of bag do you usually use to carry your books and school supplies?

3.1.1.	Backpack	
3.1.2.	Handbag on a short strap that can be worn over the shoulder	
3.1.3.	Backpack on wheels	
3.1.4.	Briefcase/bag without an ear	
3.1.5.	Bag on a long strap that can be slung over the shoulder	
3.1.6.	Other (What kind)	

		Never	Almost never	Almost always	Always
3.2.	I carry heavy items in my backpack.				
3.3.	When packing my backpack, I only put the essentials in it.				
3.4.	I put the backpack on both shoulders. 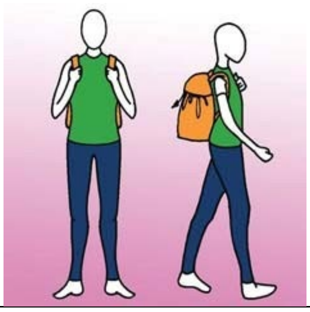				
3.5.	When packing the backpack, I distribute the weight of the books so that the heaviest ones are close to my back. 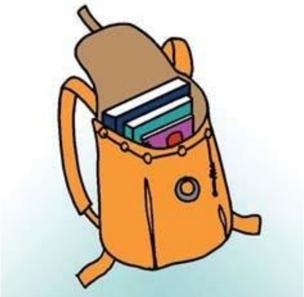				
3.6.	When carrying a lot of weight, I use a stroller or backpack on wheels, not a backpack carried on my shoulders. 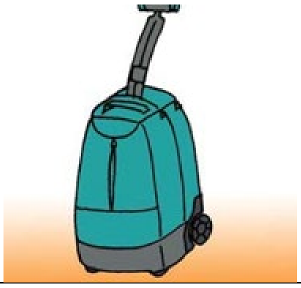				

3.7.	When carrying a heavy load, I push a stroller or backpack on wheels instead of pulling it.<br>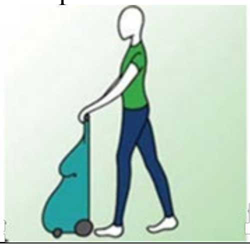 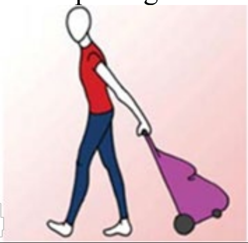				
3.8.	When carrying or holding a heavy weight in my hands, I do it with my arms bent and close to my body.<br>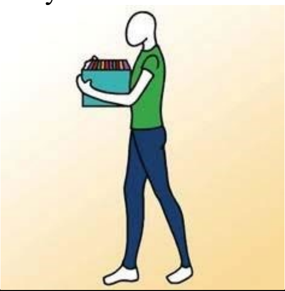				
3.9.	When carrying heavy bags, I try to distribute the weight evenly on both shoulders.<br>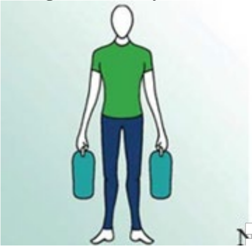 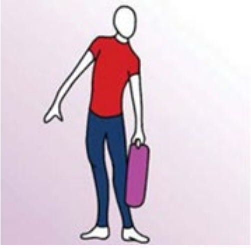				
3.10.	When lifting a weight from the ground, I keep my back straight and transfer the weight to my legs.<br>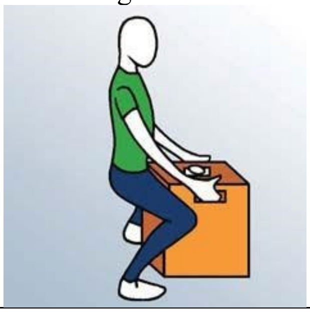				
3.11.	When picking up an object that is above my head, I climb the ladder/stairs until it is in front of me.<br>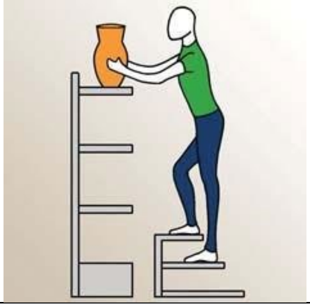				
3.12.	When I have to lift a heavy weight off the ground, I usually ask for help.				

	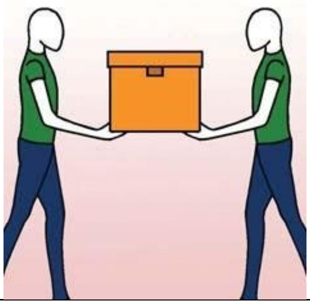				
3.13.	When picking up an object from the ground that is next to me, I bend and turn my back to pick it up. 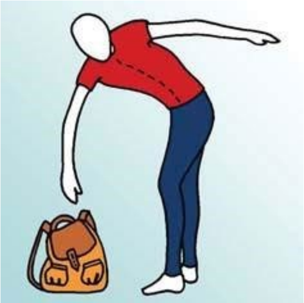				

4. WHEN I'M LYING:

I usually sleep:

		Never	Almost never	Almost always	Always
4.1.	On my stomach.				
4.2.	In the embryonic position.				
4.3.	On my back.				
4.4.	The mattress I have on my bed is soft.				
4.5.	The mattress I have on my bed is hard.				

Filing date: ____________________________

Thank you for your cooperation!
