# Supplementary material for: Cross-Cultural Validity and Reliability of the Questionnaire on Back-Health-Related Postural Habits During Daily Activities in the Polish Young Adolescent Population
Source: J Clin Med. 2025 Nov 3;14(21):7793. doi: 10.3390/jcm14217793 (PMC12608106; doi:10.3390/jcm14217793)
Supplement: Supplementary file 1 [file jcm-14-07793-s001.zip › File S2.pdf]

WIEK ……………………..

Dobrowolnie wyrażam świadomą zgodę na udział w badaniu naukowym oraz prezentację wyników na konferencjach oraz w publikacjach naukowych.

O WYRAŻAM ZGODĘ **O NIE WYRAŻM ZGODY**

KWESTIONARIUSZ DBANIA O KRĘGOSŁUP I NAWYKÓW ZWIĄZANYCH Z POSTAWĄ CIAŁA

ZAZNACZ IKSEM (X) ODPOWIEDŹ, KTÓRĄ UWAŻASZ ZA NAJBARDZIEJ ODPOWIEDNIĄ

1. KIEDY STOJĘ:

		Nigdy	Prawie nigdy	Prawie zawsze	Zawsze
1.1.	Kiedy stoję i myję zęby, podtrzymuję się wolną ręką, aby odciążyć pochylony kręgosłup. 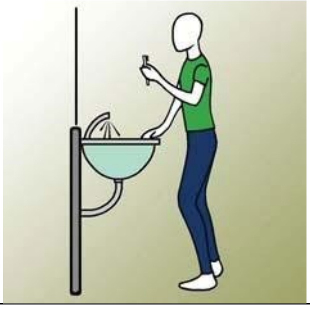				
1.2.	Kiedy stoję przez długi czas, ciągle zmieniam pozycję, przenosząc ciężar ciała z jednej nogi na drugą.				
1.3.	Kiedy stoję i wykonuję prace domowe (zamiatanie, ścielenie łóżka) staram się mieć wyprostowane plecy. 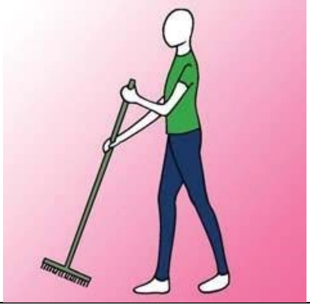				
1.4.	*To pytanie skierowane jest tylko do osób, które regularnie chodzą w butach na podwyższonych obcasach. Jeśli to pytanie Cię nie dotyczy przejdź do kolejnego punktu.*				

	Odczuwam dolegliwości bólowe kręgosłupa kiedy chodzę długo w butach na wysokim obcasie				

2. KIEDY SIEDZĘ:

		Nigdy	Prawie nigdy	Prawie zawsze	Zawsze
2.1.	Kiedy siedzę, opieram całe plecy o oparcie krzesła. 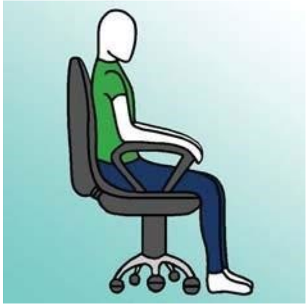				
2.2.	Kiedy siedzę, nie opieram obu stóp o podłoże. 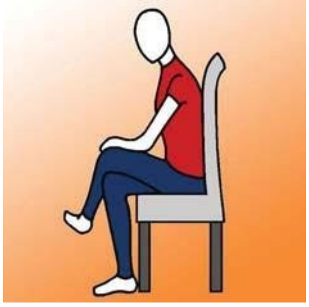				
2.3.	Kiedy siedzę, opieram pośladki na krawędzi siedzenia. 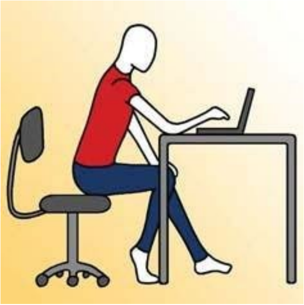				
2.4.	Kiedy siedzę, pochylam się do przodu i wyginam plecy w łuk. 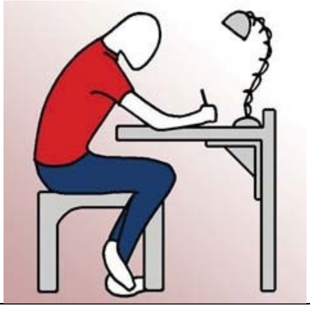				

2.5.	Kiedy siedzę, obracam tułów, żeby podnieść jakiś przedmiot lub porozmawiać z kolegami z klasy. 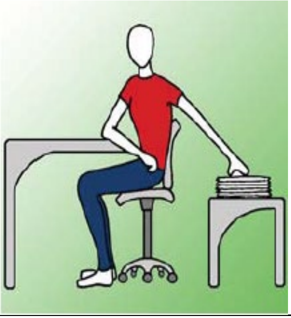				
2.6.	Kiedy siedzę, opieram przedramiona na blacie stołu. 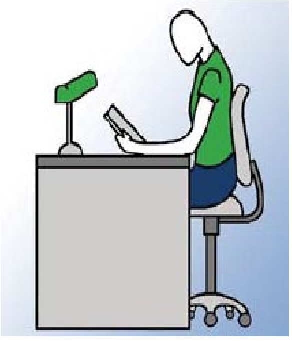				
2.7.	Kiedy siedzę, używam podpórki na książki lub tablet. 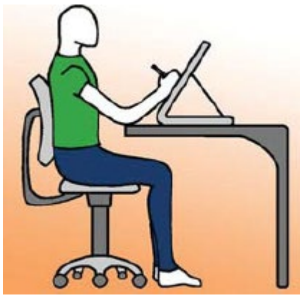				
2.8.	Kiedy pracuję na komputerze, umieszczam ekran na wysokości oczu. 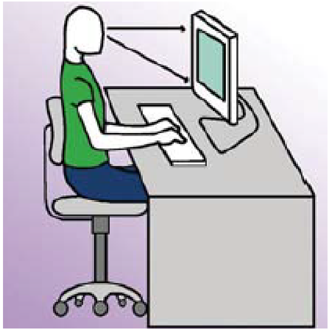				
2.9.	Kiedy pracuję na komputerze, kładę klawiaturę i mysz na wysokości łokci.				

	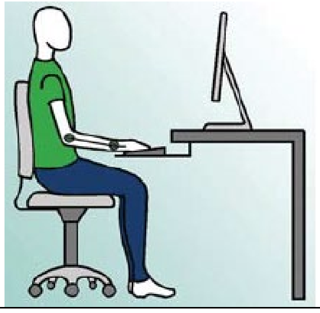				

3. KIEDY NOSZĘ CIĘŻKIE PRZEDMIOTY:

3.1. Jakiego rodzaju torby zwykle używasz do noszenia książek i przyborów szkolnych?

3.1.1.	Plecaka	
3.1.2.	Torebki na krótkim pasku, którą można założyć na ramię	
3.1.3.	Plecaka na kółkach	
3.1.4.	Teczkę/aktówkę bez ucha	
3.1.5.	Torbę na długim pasku, którą można przewiesić przez ramię	
3.1.6.	Inne (Jakiego rodzaju) ……………………………………………	

		Nigdy	Prawie nigdy	Prawie zawsze	Zawsze
3.2.	Noszę ciężkie przedmioty w plecaku.				
3.3.	Kiedy pakuję plecak, wkładam do niego tylko niezbędne rzeczy.				
3.4.	Zakładam plecak na oba ramiona. 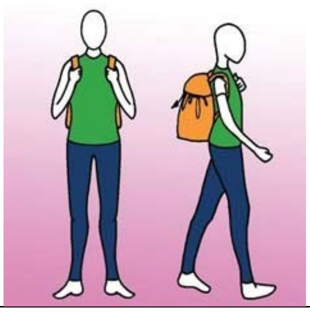				
3.5.	Kiedy pakuję plecak, rozkładam ciężar książek tak, aby najcięższe znajdowały się blisko pleców.				

	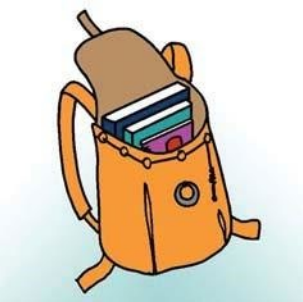				
3.6.	Kiedy dźwigam duży ciężar, używam wózka lub plecaka na kółkach, a nie plecaka noszonego na ramionach.<br>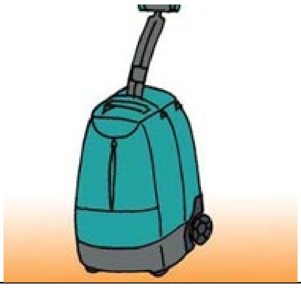				
3.7.	Kiedy dźwigam duży ciężar, pcham wózek lub plecak na kółkach zamiast go ciągnąć.<br>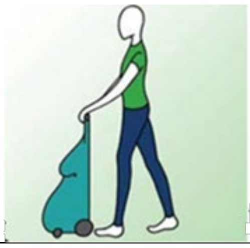 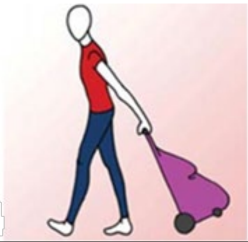				
3.8.	Kiedy niosę lub trzymam w rękach duży ciężar, robię to ze zgiętymi ramionami i blisko ciała.<br>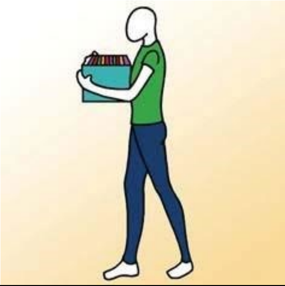				
3.9.	Kiedy noszę ciężkie torby, staram się równomiernie rozłożyć ciężar na oba ramiona.<br>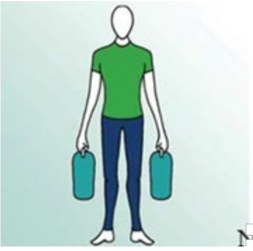 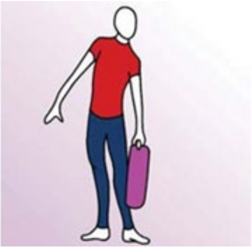				

3.10.	Kiedy podnoszę ciężar z ziemi, trzymam plecy prosto i przenoszę ciężar na nogi. 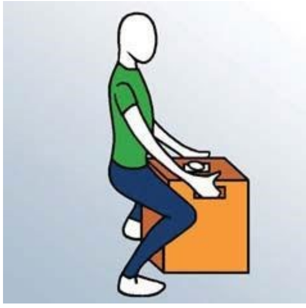				
3.11.	Kiedy podnoszę przedmiot znajdujący się nad moją głową, wspinam się po drabinie/schodkach, aż znajdzie się on przede mną. 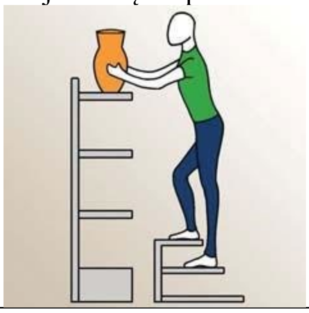				
3.12.	Kiedy muszę podnieść duży ciężar z ziemi, zwykle szukam pomocy. 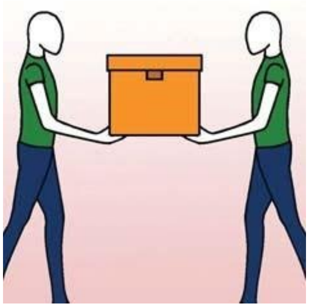				
3.13.	Kiedy podnoszę z ziemi przedmiot, który znajduje się obok mnie, zginam się i obracam plecami, aby go podnieść. 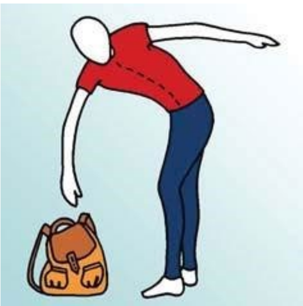				

4. KIEDY LEŻĘ:

Zwykle śpię:

		Nigdy	Prawie nigdy	Prawie zawsze	Zawsze
4.1.	Na brzuchu.				
4.2.	W pozycji embrionalnej.				
4.3.	Na plecach.				
4.4.	Materac, który mam na łóżku, jest miękki.				
4.5.	Materac, który mam na łóżku jest twardy.				

Data wypełnienia: ____________________

Dziękuję bardzo za współpracę.
